# Corollary discharges and fatigue-related symptoms: the role of attentional focus

**DOI:** 10.3389/fpsyg.2015.01002

**Published:** 2015-07-14

**Authors:** Marcelo Bigliassi

**Affiliations:** Department of Life Sciences, Brunel University LondonMiddlesex, UK

**Keywords:** fatigue, attention, exercise, brain, sensory aids

## Theoretical backdrop

Why do we stop? This question has intrigued researchers and exercise professionals worldwide (Noakes, 2000; Marcora, 2010; Amann et al., 2013). The answer is apparently linked to peripheral and cortical changes (Gandevia, 2001). Interestingly, most researchers are uncertain about the real mechanisms that underlie fatigue and task disengagement during exercise (Shephard, 2009). Due to the fact that fatigue-related symptoms are connected to psychophysiological changes and exercise performance, this question is paramount to understand the exercising human body (Marcora, 2008). Compelling evidence suggests that both peripheral changes and cortical activity create the sense of effort (Noakes, 2011). In such instance, the magnitude imposed by both cortical and peripheral changes will be determined in accord with the exercise mode and intensity. The present piece of work aims to provide an update on the psychobiological model (Pageaux, 2014) based on the evidence that attentional focus manipulates exerciser's consciousness with consequent effects on exertional responses and exercise performance (Lohse et al., 2010; Lohse and Sherwood, 2011). “The psychobiological model is an effort-based decision making model based on motivational intensity theory, and postulates that the conscious regulation of pace is determined primarily by five different cognitive/motivational factors: Perception of effort; potential motivation; knowledge of the distance/time to cover; knowledge of the distance/time remaining; previous experience/memory of perception of effort during exercise of varying intensity and duration” (Pageaux, 2014). An integrative model has been developed to underpin psychological responses that occur in response to the increasing exercise intensity.

## When the brain decides to stop

There must be a purpose for animals to act the way they do (Darwin, 1859). The brain commands the whole body through a sequence of neural outputs. When humans do not want to exercise, they simply stop, meaning that motivation plays a central role in exercise engagement (Marcora, 2008). However, there are physical and mechanical limits imposed by the human structure. Motivational stimuli such as music and video have been extensively used in the field of sport and exercise as a means by which to increase situational motivation with consequent impact upon exercise performance (Karageorghis et al., 2013; Hutchinson et al., 2015). When sensory strategies are used during exercise tasks, the exerciser initially reallocates his/her attentional focus to the stimulus. This attentional switching caused by the relevance of the stimulus can modulate psychophysiological variables over time (Razon et al., 2009). In such application, the sensory stimulus competes for attention (Rejeski, 1985); therefore, the human brain selects the most relevant signals and gives these the most attention (Treisman, 1964). The increasing exercise intensity forces the brain to focus on the signals of exertion, because those are considered as more relevant to finish the trial successfully (Hutchinson and Tenenbaum, 2007; Wittekind et al., 2011).

Afferent feedback from visceral organs are theorized to influence attentional focus by increasing exertional responses (Hutchinson and Karageorghis, 2013). In such instance, sensory strategies such as auditory and visual stimuli are capable of ameliorating fatigue-related symptoms by competing for attention (Hutchinson et al., 2015). However, compelling evidence indicates that exertion might be not influenced by afferent feedback; patients with transplanted heart reported similar rate of perceived exertion (RPE) during exercise-related situations (Braith et al., 1992). The same results have been previously reported with epidural anesthesia, which blocks afferent signals from the working muscles (Fernandes et al., 1990), meaning that cortical activity is certainly associated with exertional responses and may be only partially connected to peripheral changes (Pollak et al., 2014). In this case, the central motor command apparently emits parallel messages (corollary discharges) to the brain regions associated with exertion as a means by which to create the sense of effort (De Morree et al., 2014).

In order to demonstrate that corollary discharges are responsible for influencing RPE, researchers have used hypnosis to recreate the exercise sensations (Williamson et al., 2002). In such application, exertional responses are theorized to be exclusively representative of cortical activity because participants were comfortably still. Williamson et al. (2001) used hypnosis to recreate the sensations of cycling downhill and uphill. When participants pictured themselves in such situations, they reported greater exertion during the uphill condition. This study demonstrated that the brain regions associated with exertion are at least partially influenced by cortical activity, because participants did not achieve maximal levels of exertion.

During exercise conditions, psychophysical phenomena such as motivation, perception of effort, and sensory information act upon voluntary control and neural activation of the working muscles (McCormick et al., 2015). Corollary discharges are hypothesized to be sent from the central motor command to many brain regions; those signals regulate perceived exertion, which negatively influences exercise engagement (De Morree et al., 2012). However, corollary signals can also act upon motivation and self-belief. In this case, corollary signals assume inhibitory characteristics, which decrease neural activation by making perceived exertion the only variable capable of influencing voluntary control.

It is also important to point out that conscious processes can generate positive signals to compete with corollary signals. In other words, people can resist this negative influence through conscious motivation (e.g., self-talk; see Blanchfield et al., 2014). It is noteworthy that awareness and sensory information can also have negative influences on voluntary control; for example, if the exerciser engages in negative self-talk or listens to unpleasant music while running. Those negative influences are theorized to decrease motivational state and increase perceived exertion. In addition, brain responses to high levels of anxiety are also responsible for increasing perception of effort and decreasing voluntary control with negative influence on performance (Parry et al., 2011). Therefore, it is postulated that a greater amount of corollary discharge might be sent to sensory regions of the brain, reducing efferent signals, and muscle activity.

## The role of attentional focus

The current piece of work postulates that attentional focus is the trigger responsible for modulating psychological responses to exercise. In such instance, the exerciser initially reallocates his/her attentional focus to the stimulus considered more relevant (Broadbent, 1958; Razon et al., 2012). Exercisers are able to focus on task-unrelated cues at low-intensity exercise, because the exercise task is considerably easy (for details, see Hutchinson and Tenenbaum, 2007). As soon as the exercise intensity increases, the human brain reallocates the attentional focus to internal sensory cues such as corollary discharges. This process occurs several times throughout the exercise cycle depending on the stimulus relevance (Tenenbaum and Connolly, 2008). Hence, attentional focus controls the exerciser's awareness to associative or dissociative thoughts, with subsequent impact upon exertional responses and exercise performance (see Ille et al., 2013).

**Figure 1:**
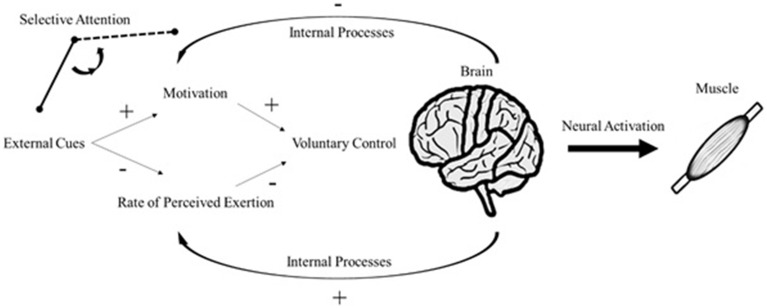
**An update on the psychobiological model of endurance performance**. Plus signs represent a positive effect on the subsequent response; conversely, minus signs represent a negative effect on the subsequent response (e.g., internal processes decrease motivation and increase RPE).

Attentional focus is manipulated according to the signal relevance (Treisman, 1964). In such instance, sensory stimulation can reallocate attention to task-unrelated cues such as auditory and visual stimuli (Loizou and Karageorghis, 2015). If the sensory stimulus receives greater attention, the processing of corollary signals is attenuated, because the brain has limited capacity to process internal and external sensory cues (Rejeski, 1985). The distractive response induced by the sensory stimulus is capable of enhancing situational motivation and ameliorating the effects of fatigue-related symptoms even at high-intensity exercises (Boutcher and Trenske, 1990; Jones et al., 2014). Regardless of the source of internal processes (corollary discharges or afferent feedback), environmental influences compete for attention throughout the exercise cycle. Therefore, selective attention between external and internal sensory cues is in charge of initiating cascade reactions to psychophysical responses (Figure 1).

## No more headless bodies walking alone

The information provided in this piece of work indicates that this field of research is hitherto under-researched. Researchers are encouraged to investigate the cortical and psychophysiological mechanisms that underlie task disengagement during whole-body modes of exercise. The answer might be associated with a constant flow of information between the brain and periphery. The psychological mechanisms that underlie task disengagement during exercise apparently interact with each other in order to define the moment at which humans stop exercising. Attentional focus appears to be the trigger responsible for initiating cascade reactions that underpin the sense of effort and the neural activation to the active muscles. The exercise mode and intensity influence exertional responses over time (Razon et al., 2012). Sensory stimulation/deprivation and attentional strategies (associative or dissociative behavior) can facilitate or jeopardize exercise performance.

## Conflict of interest statement

The author declares that the research was conducted in the absence of any commercial or financial relationships that could be construed as a potential conflict of interest.
